# Improving health literacy and stakeholder-directed knowledge of One Health through analysis of readability: a cross sectional infodemiology study

**DOI:** 10.1016/j.soh.2024.100088

**Published:** 2024-11-07

**Authors:** John E. Moore, Beverley C. Millar

**Affiliations:** a Laboratory for Disinfection and Pathogen Elimination Studies, Northern Ireland Public Health Laboratory, Belfast City Hospital, Lisburn Road, Belfast, BT9 7AD, Northern Ireland, UK; b School of Medicine, Dentistry and Biomedical Sciences, The Wellcome-Wolfson Institute for Experimental Medicine, Queen's University, 97 Lisburn Road, Belfast BT9 7BL, Northern Ireland, UK; c School of Biomedical Sciences, Ulster University, Cromore Road, Coleraine, Co. Londonderry, Northern Ireland, BT52 1SA, UK

**Keywords:** One Health, Readability, Health literacy, Flesch Reading Ease, Flesch-Kincaid Grade Level

## Abstract

**Background:**

The One Health approach involves collaboration across several sectors, including public health, veterinary and environmental sectors in an integrated manner. These sectors may be disparate and unrelated, however to succeed, all stakeholders need to understand what the other stakeholders are communicating. Likewise, it is important that there is public acceptance and support of One Health approaches, which requires effective communication between professional and institutional organisations and the public. To help aid and facilitate such communication, written materials need to be readable by all stakeholders, in order to communicate effectively. There has been an exponential increase in the publication of papers involving One Health, with <5 per year, in the 2000s, to nearly 500 published in 2023. To date, readability of One Health information has not been scrutinised, nor has it been considered as an integral intervention of One Health policy communication. The aim of this study was therefore to examine readability of public-facing One Health information prepared by 24 global organisations.

**Methods:**

Readability was calculated using *Readable* software, to obtain four readability scores [(ⅰ) Flesch Reading Ease (FRE), (ⅱ) Flesch-Kincaid Grade Level (FKGL), (ⅲ) Gunning Fog Index and (ⅳ) SMOG Index] and two text metrics [words/sentence, syllables/word] for 100 sources of One Health information, from four categories [One Health public information; PubMed abstracts; *Science in One Health* (SOH) abstracts (articles); SOH abstracts (reviews)].

**Results:**

Readability of One Health information for the public is poor, not reaching readability reference standards. No information was found that had a readability of less than 9th grade (around 14 years old). Mean values for the FRE and FKGL were (19.4 ± 1.4) (target >60) and (15.6 ± 0.3) (target <8), respectively, with mean words per sentence and syllables per word of 20.5 and 2.0, respectively. Abstracts with “One Health” in the title were more difficult to read than those without “One Health” in the title (FRE: *P* = 0.0337; FKGL: *P* = 0.0087). Comparison of FRE and FKGL readability scores for the four categories of One Health information [One Health public information; PubMed abstracts; SOH abstracts (articles); SOH abstracts (reviews)] showed that SOH abstracts from articles were easier to read than those from SOH reviews. No One Health public-facing information from the 100 sources examined met the FKGL target of ≤8. The most easily read One Health information required a Grade Level of 9th grade (14–15 years old), with a mean Grade Level of 15.5 (university/college level).

**Conclusion:**

Considerable work is required in making One Health written materials more readable, particularly for children and adolescents (<14 years of age). It is important that any interventions or mitigations taken to support better public understanding of the One Health approach are not ephemeral, but have longer lasting and legacy value. Authors of One Health information should consider using readability calculators when preparing One Health information for their stakeholders, to check the readability of their work, so that the final material is within recommended readability reference parameters, to support the health literacy and stakeholder-directed knowledge of their readers.

## 1 Introduction

The World Health Organization (WHO) defines One Health as an integrated, unifying approach to balance and optimize the health of people, animals and the environment, which mobilises multiple sectors, disciplines and communities at varying levels of society to work together, to create long-term, sustainable solutions [1].

The One Health approach involves collaboration across several sectors, including public health, veterinary and environmental sectors, in an integrated manner. These sectors may be disparate and unrelated, however to succeed, all stakeholders need to understand what the other stakeholders are communicating. Each of these three sectors have evolved their own lexicon and specialist language, which maybe is not that well understood by other disciplines. Likewise, it is important that there is public acceptance and support of One Health approaches, which requires effective communication between professional and institutional organisations and the public. To help aid and facilitate such communication, written materials need to be readable by all stakeholders, in order to communicate effectively.

Readability is a measure of how easy a piece of text is to read and has become an important measurable parameter within healthcare, particularly when trying to assess how well patient-facing written material has been prepared. Readability is assessed using established evidence-based formulae, quantifying words per sentence, syllables per word, as well as other syntax parameters. Readability formulae commonly quoted in healthcare analysis include the Flesch-Kincaid Grade Level (FKGL) and the Flesch Reading Ease (FRE) scores [2] (Supplementary Table 1). To date, there has not been any analysis conducted that has examined the readability of public-facing materials describing One Health. If One Health information has poor readability, then the public, as well as non-specialist stakeholders may be less likely to understand the narrative, which may result in poor One Health knowledge and policy decisions. A good understanding of One Health information is therefore vital for an individual or organisations to adequately comprehend, in a way to maximise knowledge and understanding of One Health, as well as helping to inform and aid in the creation of policy decisions.

The aim of this study was therefore to examine the readability of publicly-facing One Health information from multiple stakeholder authors, to establish how readable public-facing One Health information is, compared to health-literacy readability reference standards, as well as to compare One Health readability with that from scientific information, from non-One Health sources, as a suitable control.

## 2 Methods

**Fig. 1:**
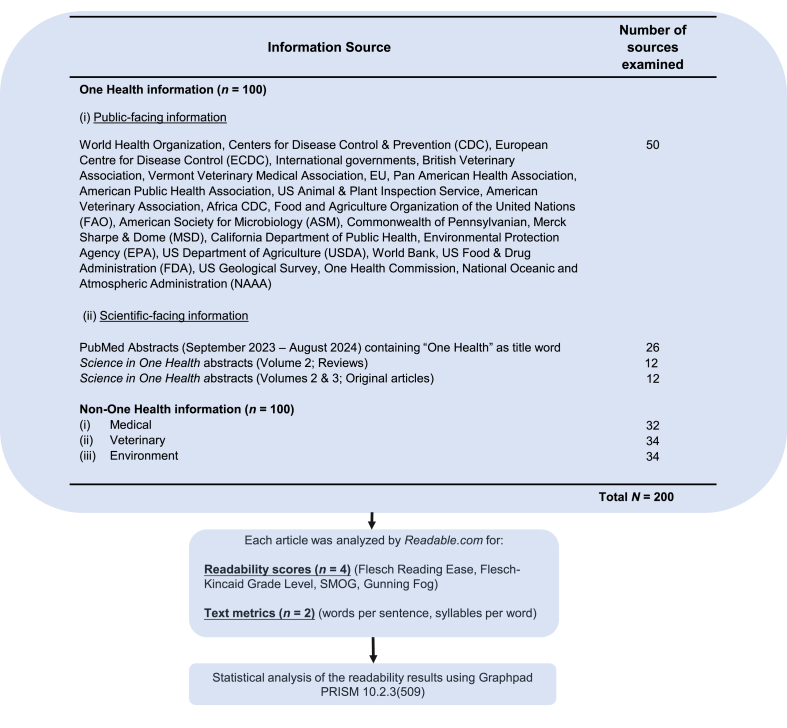
Flow diagram of methodological investigations undertaken in this study and sources of One Health information. Abbreviation: SMOG, simple measure of gobbledygook.

An overview of the sources of One Health information and the methods employed is shown in Fig. 1.

### 2.1 Retrieval of information on One Health

Information on One Health (*n* = 100 sources) was obtained from (ⅰ) public-facing information sources, which were publicly and freely available web resources, within the public domain (*n* = 50; 24 institutional sources) and (ⅱ) scientific-facing information (*n* = 50) taken from PubMed abstracts and abstracts from *Science in One Health* (SOH) reviews and original articles, which were freely available and in the public domain.

### 2.2 Retrieval of information on control (non-One Health) information sources

Information on non-One Health (*n* = 100 sources) was obtained and used as a control, for comparison with similar information from One Health sources (*n* = 100 sources). The three pillars of the One Health concept, namely human medicine, veterinary medicine and the environment were selected. In these control groups, the source information purposefully did not include the key word “One Health” in the title of the information sources examined. Three categories of non-One Health information was selected, including (ⅰ) human medicine and infection/microbiology (*n* = 32), (ⅱ) veterinary sources (*n* = 34) and (ⅲ) environment sources (*n* = 34). These information sources comprised of scientific abstracts and “other information”. Scientific abstracts (at least *n* = 20 in each group) were selected from PubMed and Google Scholar. Title word search criteria included “veterinary” or “environment” in the case of categories (ⅱ) and (ⅲ). Category (ⅰ) consisted of mainly microbiology and infectious diseases information [[3], [4], [5], [6], [7], [8], [9], [10], [11], [12], [13], [14], [15], [16], [17], [18], [19], [20], [21], [22]]. Public-facing information sources which were not related to One Health were publicly and freely available web resources, from reputable institutions and constituted at least 12 information sources.

### 2.3 Determination of readability scores and text metrics

Each source of One Health and non-One Health information was processed from its PDF or URL source, using the online subscription-based software, *Readable* (www.readable.com), which was used as guided by the manufacturer. All readability analyses were performed on text written in the English language. FRE, FKGL, Gunning Fog Index and simple measure of gobbledygook (SMOG) Index were calculated. An additional two text metrics were also calculated, including words/sentence and syllables/word, as is generally the case with such studies [23,24]. *Readable.com* was the software of choice as it has been used in several readability studies within healthcare [23,24]. McGrath and colleagues concluded that *Readable* was the best analytical tool to employ in readability studies, due to its user experience, capacity and accuracy [25].

### 2.4 Statistical analyses

Resulting data underwent statistical analyses using *GraphPad PRISM* version 10.2.3 (509) (Boston, USA). All data were tested for normality using the Kolmogorov-Smirnov test. For specific statistical analyses performed, please see footnote to figure legends. A *P* value of <0.05 was considered as statistically significant.

## 3 Results

**Fig. 2:**
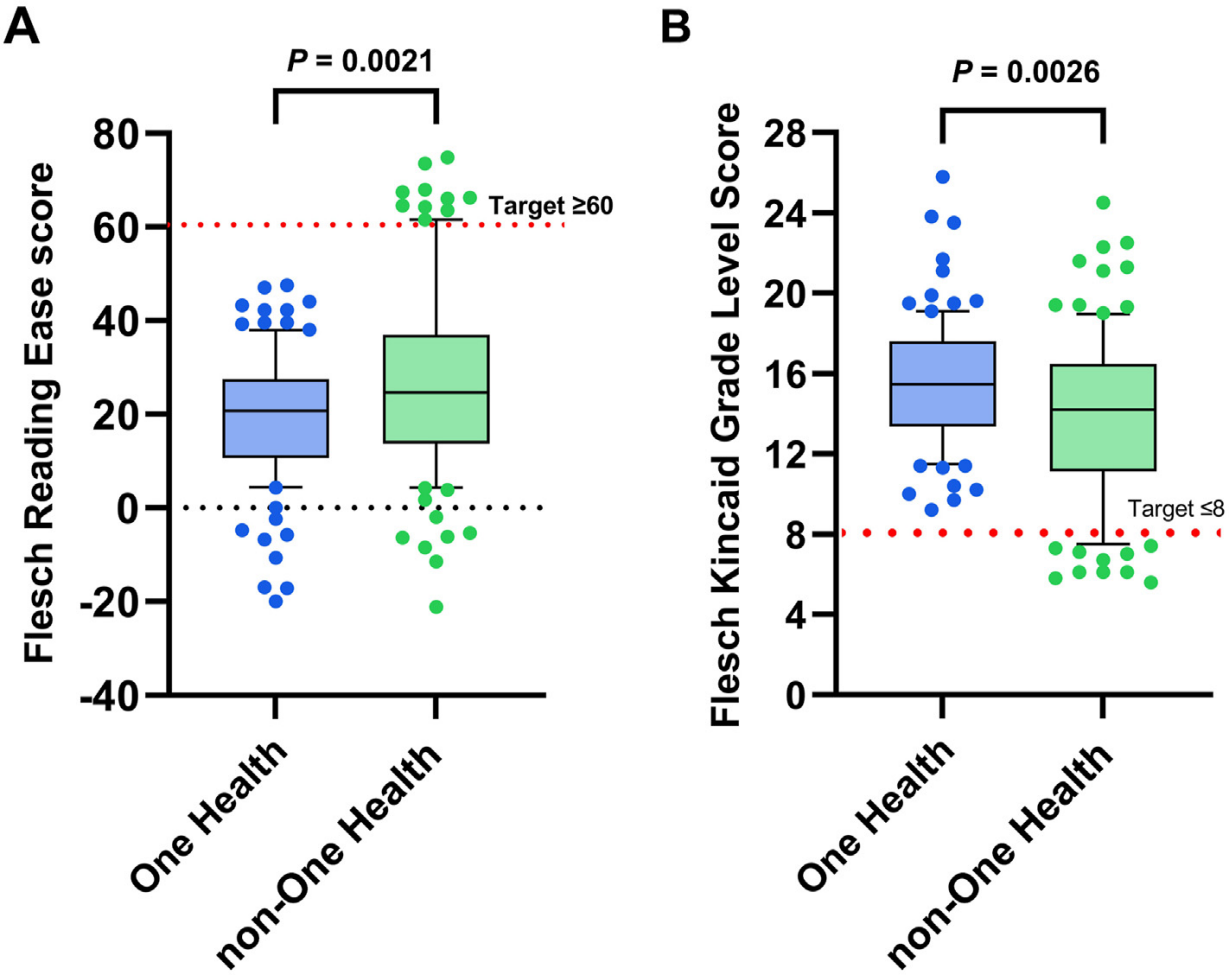
Box and whisker plot comparing readability scores calculated on total combined One Health information (*n* = 100 sources) and non-One Health information (*n* = 100 sources). (A) Flesch Reading Ease; (B) Flesch-Kincaid Grade Level. Box represents 25th and 75th percentile and bar represents the median. Whiskers represent the 10th and 90th percentile and scattered dots represent outliers outside these percentile ranges. The dashed red line represents target readability scores. For the Flesch Reading Ease, the target score is ≥ 60. For the Flesch-Kincaid Grade Level, the target score is ≤ 8. Statistical significance was calculated using an unpaired *t*-test (parametric). *P* < 0.05 was considered as statistically significant.

Readability scores for all 100 combined One Health information sources and non-One Health information, for the FRE, the FKGL, the Gunning Fog score and the SMOG score, are shown in Fig. 2. Mean values ± standard error of mean (SEM) for the FRE and FKGL for One Health information were (19.4 ± 1.4) (target >60) and (15.6 ± 0.3) (target <8), respectively, with mean words per sentence and syllables per word of 20.5 and 2.0, respectively.

Descriptive statistics for (ⅰ) One Health information and (ⅱ) non-One Health information, is shown in Table 1.

**Table 1:** Descriptive statistics of the readability of One Health information and non-One Health information.

Statistics	Gunning Fog Index	SMOG Index	Words/sentence	Syllables/word
One Health information (*n* = 100)				
Minimum	10.0	11.8	9.2	1.6
25th percentile	15.3	15.2	16.2	1.9
Median	18.2	16.9	19.2	2.0
75th percentile	20.1	18.6	25.2	2.0
Maximum	29.2	25.1	39.3	2.4
Range	19.2	13.3	30.1	0.8
Mean	17.9	16.8	20.5	2.0
Standard deviation	3.76	2.59	6.28	0.14
Standard error of mean	0.38	0.26	0.63	0.01
Non-One Health information (*n* = 97)				
Minimum	7.7	8.9	5.4	1.4
25th percentile	13.3	13.4	13.6	1.8
Median	16.5	15.8	17.8	1.9
75th percentile	19.6	17.7	23.8	2.0
Maximum	26.6	22.3	34.8	2.4
Range	18.9	13.4	29.4	1.0
Mean	16.4	15.5	18.8	1.9
Standard deviation	4.50	3.24	6.42	0.19
Standard error of mean	0.46	0.33	0.65	0.02

Abbreviation: SMOG, simple measure of gobbledygook.

**Fig. 3:**
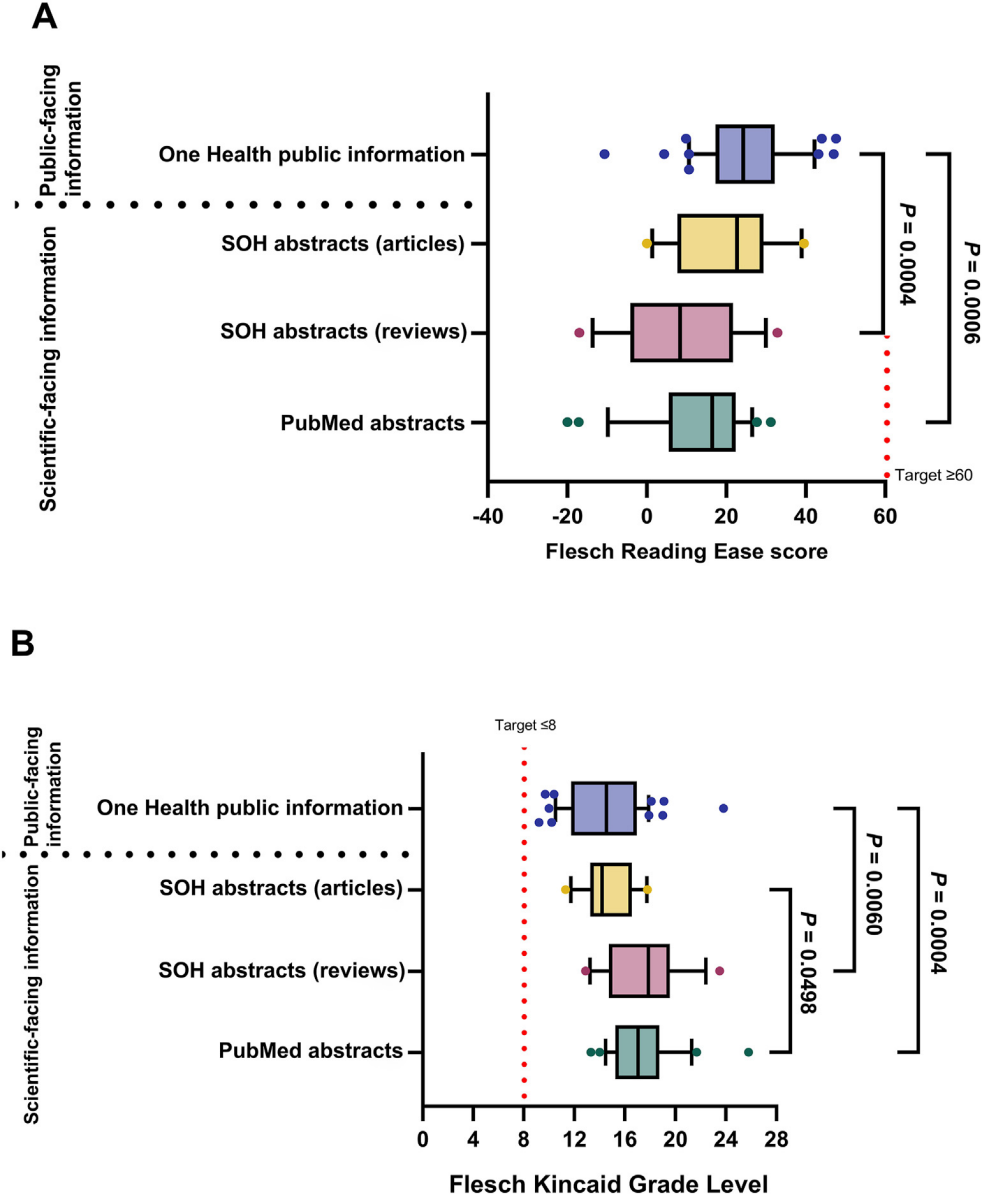
Box and whisker plot comparing readability scores of four sources of One Health information [Health public information, PubMed abstracts; *Science in One Health* (SOH) abstracts (articles); SOH abstracts (reviews)] by (A) Flesch Reading Ease and (B) Flesch-Kincaid Grade Level. Box represents 25th and 75th percentile and bar represents the median. Whiskers represent the 10th and 90th percentile and scattered dots represent outliers outside these percentile ranges. Statistical significance is shown, calculated using ANOVA (parametric) with Tukey's multiple comparisons test. *P* < 0.05 was considered as statistically significant. The dashed red line represents the target readability score. For the Flesch Reading Ease, the target score is ≥ 60. For the Flesch-Kincaid Grade Level, the target score is ≤ 8.

Comparison of FRE and FKGL readability scores for the four categories of One Health information [One Health public information; PubMed abstracts; SOH abstacts (articles); SOH abstracts (reviews)] is shown in Fig. 3.

**Fig. 4:**
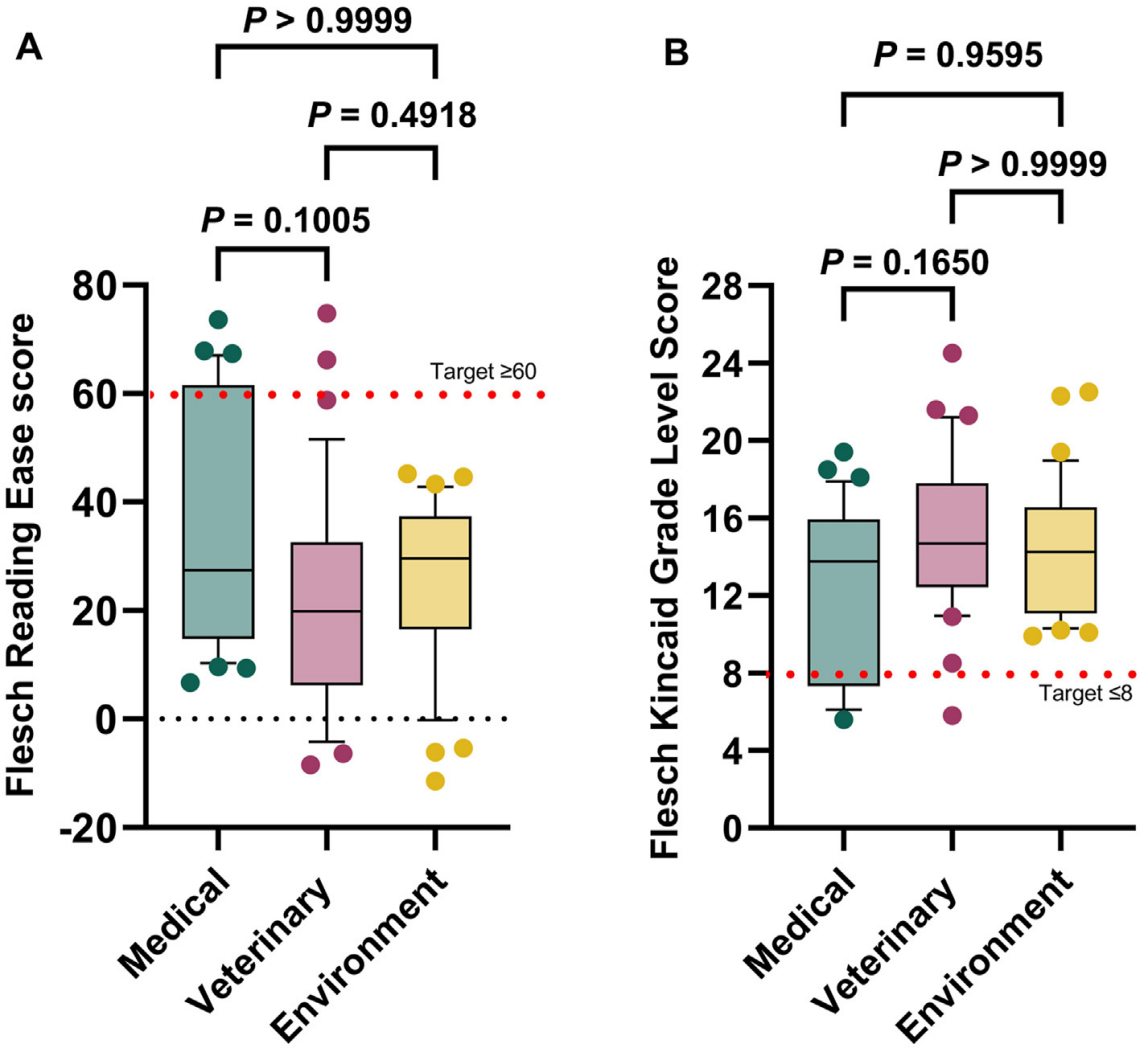
Box and whisker plot comparing readability scores comparing (ⅰ) medical (n = 32), (ⅱ) veterinary (n = 34) and (ⅲ) environment (*n* = 34) of non-One Health information for (A) Flesch Reading Ease and (B) Flesch-Kincaid Grade Level. Box represents 25th and 75th percentile and bar represents the median. Whiskers represent the 10th and 90th percentile and scattered dots represent outliers outside these percentile ranges. Statistical non-significance is shown, calculated using a Kruskal-Wallis (non-parametric) test with Dunn's multiple comparisons. *P* < 0.05 was considered as statistically significant.

Comparison of FRE and FKGL readability scores for non-One Health information namely, (ⅰ) medical, (ⅱ) veterinary and (ⅲ) environment (without “One Health” in the title) are shown in Fig. 4. There was no significant difference (*P* > 0.05) in the readability scores for any of these three categories.

**Fig. 5:**
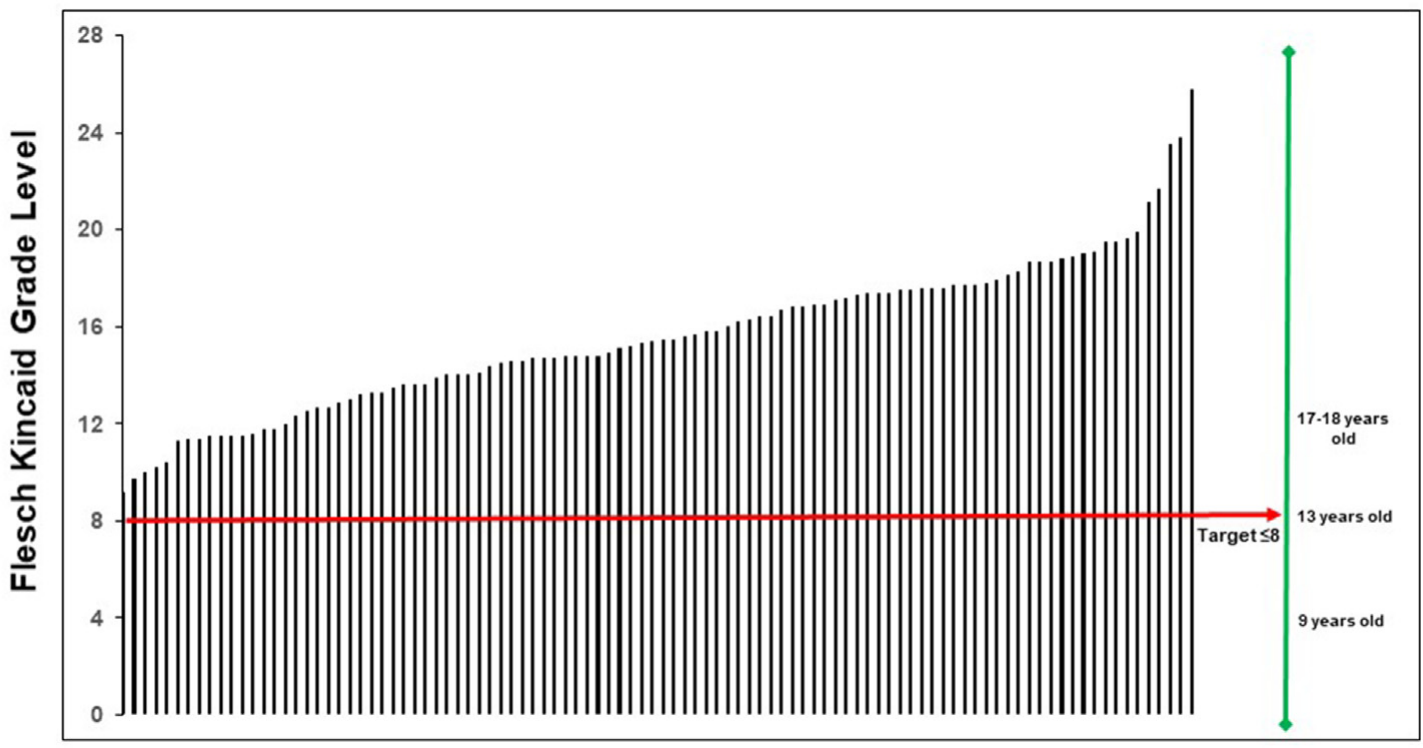
Flesch Kincaid Grade Level for One Health information, showing reference target level (≤8) and associated ages across all 100 One Health information sources.

No One Health public-facing information from the 100 sources examined met the FKGL target of ≤8 (Fig. 5). The most easily read One Health information required a Grade Level of 9th grade (14–15 years old), with a mean Grade Level of 15.5 (university/college level).

## 4 Discussion

**Fig. 6:**
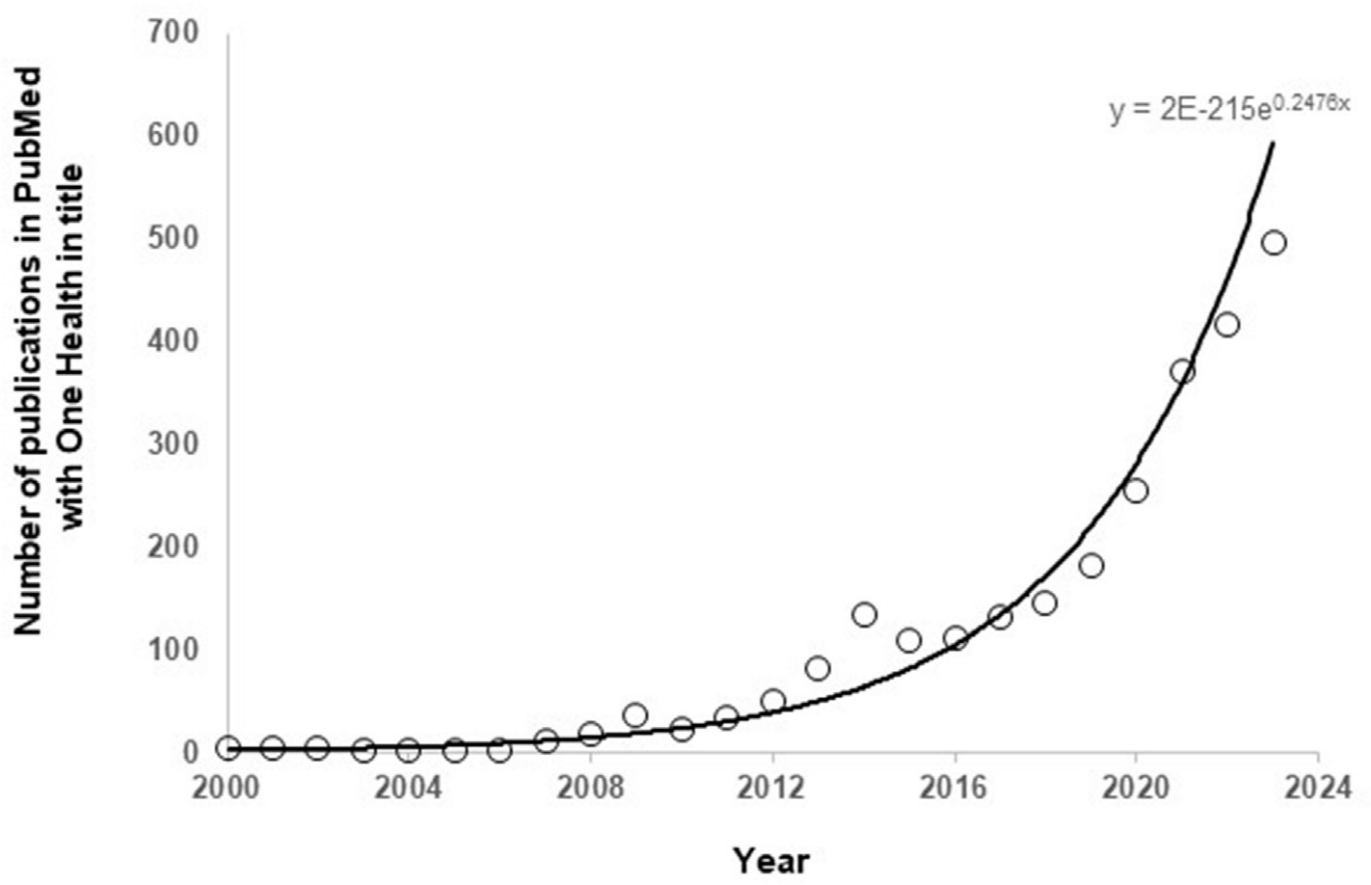
Number of publications cited by PubMed with “One Health” in title for the period 2000–2023.

Peer-reviewed scientific papers involving One Health have now risen exponentially since 2010. Prior to 2010, there were less than five publications listed per annum on PubMed, to the situation in 2023, where there were approximately 500 publications listed with “One Health” in the title (Fig. 6). Such a significant rise in the volume of written text adds to reading lists for all stakeholders with an interest or association with One Health.

To our knowledge, the current study is the first to conduct an assessment of the readability of public-, as well as scientific-facing information on One Health. Most clinical conditions have now had a readability check performed on information relating to that particular condition, to assess the quality of information for the public. For example, in a recent examination of the readability of patient education materials for heart failure in the US, of the 36 online resources examined, higher readability levels were noted than those recommended by the National Institutes of Health and the American Medical Association [26]. Likewise, a similar study examining metastatic renal cell carcinoma treatment, from the 39 websites examined, no website had a reading level of lower than 9th grade [27]. Therefore, we believe it is now timely to perform such an evaluation with One Health information and sources.

In this study, we employed quantitative measurement of words, sentences and syllables, as defined by readability formulae, including FRE, FKGL, Gunning Fog and SMOG scores (Supplementary Table 1). The design of our study involved the analyses of readability of four main sources of information on One Health, namely (ⅰ) public information from 24 institutions, (ⅱ) PubMed scientific abstracts on One Health, (ⅲ) SOH abstracts (articles) and (ⅳ) SOH abstracts (reviews). In our execution of this study, we tried to emulate the journey taken by a member of the public trying to find out more information on One Health. From examination and comparison of the readability and text metrics results of this study, the overall readability and text metric scores of all combined sources of One Health information did not meet the readability reference targets of ≥60 for the FRE score and ≤8 for the FKGL (Fig. 2), where the mean values obtained were 19.4 and 15.6, respectively. No source of public-facing information on One Health achieved the reference FRE score of ≥60 and likewise, no source reached the target FKGL score of ≤8. The American Medical Association recommends that all patient-facing material be written at a 6th grade level (11 years old) [25], whilst the Centers for Disease Control and Prevention (CDC) recommends that patient-facing information is no higher than 8th grade [25].

The current study on One Health readability represents an initiative to help address communication enhancement with the One Health approach. Another initiative to enhance communication has been the development of the One Health European Joint Programme (OHEJP) Glossary, which includes 1000 curated items covering 694 unique terms [28]. Differences in terminology and interpretation of terms are still a significant hurdle for cross-sectoral information exchange and collaboration within the area of One Health including One Health Surveillance (OHS) [28], and it is hoped that the existence of a glossary will help support cross-sectoral collaboration.

From the current study, One Health information is not well written for the public and is thus too difficult for the general public to read. Likewise, when the information sources within the four categories were compared to each other, as anticipated, the most readable of all the information sourced, was that from the 24 public information sources, which was statistically different to the scientific sources (Fig. 3). There was no significant difference in readability scores between SOH abstracts from articles and reviews. Poor readability scores were associated with higher word counts per sentence and higher syllables per word.

## 5 Study limitations

The current study has some limitations. Firstly, the One Health information collected and analysed was limited to the English language only, thereby making it of most value to people in English-speaking countries. All non-English information sources were excluded from this study, as this software cannot process language written with characters, including Chinese, Hindi and Arabic. Where English is not the first language and where countries have a high rate of illiteracy, governments, non-governmental organizations (NGOs) and public health agencies should consider an alternative to the written information and adopt alternative media, such as video, animation or podcast, to allow high quality information on One Health to be disseminated, as an alternative. Our study primarily relied on examining readability metrics such as FRE and FKGL, and did not include factors including text content, author expertise, writing style and target audience demographics, which may limit a deeper understanding of the influencing factors on readability.

Readability may be influenced by the terminology and words used, which cannot be avoided. For example, in the case of describing human infectious diseases caused by bacterial etiological agents, which are generally polysyllable, (*Actinobacillus actinomycetemcomitans*) or other organisms, e.g. *Myxococcus llanfairpwllgwyngyllgogerychwyrndrobwllllantysiliogogogochensis*, which was isolated from soil collected in the parish of Llanfairpwllgwyngyllgogerychwyrndrobwllllantysiliogogogoch, in Wales, UK [29], the need to cite the name of the organism has significant implications for the readability of the text, compared to a text describing “flu”.

More recently, in a novel suggestion by Plavén-Sigray and colleagues writing in eLife [30], these authors suggested that scientific authors should be assessed for their average readability in their writing and have suggested an “r-index”, to help promote clarity in their writing.

Readers should therefore be mindful of the potential influence of such variables on readability scores and therefore interpret such scores accordingly.

## 6 Conclusions

Readability of One Health information for the public is poor, not reaching readability reference standards. Such poor readability could be reflected in poor understandability, amongst One Health collaborators, as well as the general public. To date, readability of One Health information has not been scrutinised, nor has it been considered as an integral intervention of One Health policy communication. It is important that any interventions or mitigations taken to support better public understanding of the One Health approach are not ephemeral, but have longer lasting and legacy value. Authors of One Health information should therefore consider the adoption of readability calculators when preparing One Health written materials, as well as examine the readability of their work.

## CRediT authorship contribution statement

**John E. Moore:** Writing – review & editing, Writing – original draft, Visualization, Validation, Software, Resources, Project administration, Methodology, Investigation, Formal analysis, Data curation, Conceptualization. **Beverley C. Millar:** Writing – review & editing, Writing – original draft, Visualization, Validation, Software, Resources, Project administration, Methodology, Investigation, Formal analysis, Data curation, Conceptualization.

## Patient consent for publication

Not applicable.

## Ethics statement

This study did not involve human or animal subjects. All of the material used in this study was openly and freely available to the public and within the public domain.

## Availability of data and materials

All data supporting the findings of this report are freely available in the public domain for access by readers.

## Financial support

None. This study was supported by internal funding.

## Declaration of competing interest

The authors declare that they have no known competing financial interests or personal relationships that could have appeared to influence the work reported in this paper.
